# Post-traumatic stress disorder as a risk factor for major adverse cardiovascular events: a cohort study of a South African medical insurance scheme

**DOI:** 10.1017/S2045796024000052

**Published:** 2024-02-05

**Authors:** Cristina Mesa-Vieira, Christiane Didden, Michael Schomaker, Johannes P. Mouton, Naomi Folb, Leigh L. van den Heuvel, Chiara Gastaldon, Morna Cornell, Mpho Tlali, Reshma Kassanjee, Oscar H. Franco, Soraya Seedat, Andreas D. Haas

**Affiliations:** 1 Institute of Social and Preventive Medicine, University of Bern, Bern, Switzerland; 2 Graduate School for Health Sciences, University of Bern, Bern, Switzerland; 3 Department of Sociology, Ludwig-Maximilians-Universität Munich, Munich, Germany; 4 Department of Statistics, Ludwig-Maximilians-Universität Munich, Munich, Germany; 5 Centre for Infectious Disease Epidemiology & Research, School of Public Health, University of Cape Town, Cape Town, South Africa; 6 Division of Clinical Pharmacology, Department of Medicine, University of Cape Town, Cape Town, South Africa; 7 Medscheme, Cape Town, South Africa; 8 Department of Psychiatry, Faculty of Medicine and Health Sciences, Stellenbosch University, Cape Town, South Africa; 9 South African Medical Research Council/Stellenbosch University Genomics of Brain Disorders Research Unit, Stellenbosch University, Cape Town, South Africa; 10 Department of Global Public Health & Bioethics, University Medical Center Utrecht, Utrecht, Netherlands

**Keywords:** depression, epidemiology, health outcomes, PTSD

## Abstract

**Aims:**

Prior research, largely focused on US male veterans, indicates an increased risk of cardiovascular disease among individuals with post-traumatic stress disorder (PTSD). Data from other settings and populations are scarce. The objective of this study is to examine PTSD as a risk factor for incident major adverse cardiovascular events (MACEs) in South Africa.

**Methods:**

We analysed reimbursement claims (2011–2020) of a cohort of South African medical insurance scheme beneficiaries aged 18 years or older. We calculated adjusted hazard ratios (aHRs) for associations between PTSD and MACEs using Cox proportional hazard models and calculated the effect of PTSD on MACEs using longitudinal targeted maximum likelihood estimation.

**Results:**

We followed 1,009,113 beneficiaries over a median of 3.0 years (IQR 1.1–6.0). During follow-up, 12,662 (1.3%) persons were diagnosed with PTSD and 39,255 (3.9%) had a MACE. After adjustment for sex, HIV status, age, population group, substance use disorders, psychotic disorders, major depressive disorder, sleep disorders and the use of antipsychotic medication, PTSD was associated with a 16% increase in the risk of MACEs (aHR 1.16, 95% confidence interval (CI) 1.05–1.28). The risk ratio for the effect of PTSD on MACEs decreased from 1.59 (95% CI 1.49–1.68) after 1 year of follow-up to 1.14 (95% CI 1.11–1.16) after 8 years of follow-up.

**Conclusion:**

Our study provides empirical support for an increased risk of MACEs in males and females with PTSD from a general population sample in South Africa. These findings highlight the importance of monitoring cardiovascular risk among individuals diagnosed with PTSD.

## Introduction

Post-traumatic stress disorder (PTSD) is a persistent and impairing mental disorder that emerges following a traumatic event (O’Donnell *et al.*, 2021). The global lifetime prevalence of PTSD among the general population is estimated at 3.9%, with variations across different countries (Kessler *et al.*, 2017). In South Africa, the lifetime prevalence is estimated at 2.3% (Herman *et al.*, 2009).

The current scientific literature suggests a higher incidence of cardiovascular disease (CVD) among persons diagnosed with PTSD (Edmondson *et al*. 2013; Edmondson and von Kanel, 2017; Jacquet-Smailovic *et al*., 2022). In a meta-analysis by Edmondson *et al*., (2013), PTSD was associated with coronary heart disease. In individuals with PTSD, the pooled risk increased by 55% (95% confidence interval (CI) 34%–79%) when adjusting for sociodemographic and cardiovascular factors and by 27% (95% CI 8%–49%) after further adjustment for depression. Similarly, Jacquet-Smailovic *et al*., (2022) conducted a systematic review and meta-analysis of 14 studies to estimate the association between PTSD and the risk of subsequent myocardial infarction. Before adjusting for depression, the pooled risk increased by 49% (95% CI 31%–69%), while after adjustment, it decreased to 32% (95% CI 12%–56%).

A key limitation of prior research on the relationship between PTSD and CVD is its primary focus on males from the United States. Much of the research has focused on male veterans from the US and World Trade Center survivors, with only a few studies looking at females in the US or other high-income countries (Edmondson *et al.*, 2013; Edmondson and von Kanel, 2017; Jacquet-Smailovic *et al.*, 2022). This focus leaves a considerable knowledge gap concerning females and populations from low- and middle-income settings, who are exposed to a range of traumas that extend beyond war-related experiences. According to a nationally representative household survey conducted between January 2002 and June 2004, the most common traumatic event in South Africa is the unexpected death of a loved one, affecting 39.2% of the population, followed by physical violence at 37.6%, accidents at 31.9% and witnessing traumatic events at 29.5%. Among specific types of events, being mugged or threatened with a weapon is relatively common, with a prevalence of 18.3%. Life-threatening illnesses also affect 13.2% of the population. Sexual violence affects 7.6% of the population, with specific events such as rape and sexual assault affecting 2.1% and 1.6%, respectively (Atwoli *et al.*, 2013). However, it is important to consider that these estimates from the household survey might not reflect the full extent of the issue, given that sexual violence might be under-reported in such surveys. The Global Database on Violence Against Women reports that lifetime physical and/or sexual intimate partner violence in South Africa is at 21.3% (NDoH *et al.*, 2019), indicating that the actual prevalence of sexual violence could be considerably higher than what is suggested by the survey data.

Another potential issue with previous studies may be the inappropriate adjustment for psychiatric comorbidities. Psychiatric comorbidities which include substance use disorders (Akasaki and Ohishi, 2020), major depressive disorder, psychotic disorders (Lambert *et al.*, 2022) and sleep disorders (McCall *et al.*, 2019; Nambiema *et al.*, 2023) can be risk factors for both CVD and PTSD (Sareen, 2014). For this reason, it is necessary to adjust for these confounding factors (Hernán and Robins, 2020). However, psychiatric comorbidities can also act as mediators in the relationship between PTSD and CVD and lie on the causal pathway between exposure and outcome (Breslau *et al.*, 1997; Radell *et al.*, 2020). In such a situation, adjustment for psychiatric comorbidity using traditional survival models can lead to biased estimates (Mansournia *et al.*, 2017; Schisterman *et al.*, 2009).

In our study, we aim to quantify the increase in the risk of incident major adverse cardiovascular events (MACEs) in South African males and females diagnosed with PTSD, applying both traditional survival analysis and modern causal inference methods. South Africa, as a middle-income country with a high prevalence of diverse trauma exposures, provides a unique study setting that broadens our understanding of the PTSD-MACE relationship beyond the predominantly male, US-centric populations typically studied. Addressing the methodological limitations of previous research, we used longitudinal targeted maximum likelihood estimation. This modern causal inference approach can handle time-varying confounding affected by prior exposure without blocking the causal path from exposure to outcome (Petersen *et al.*, 2014), thereby allowing for sound adjustment for psychiatric comorbidities and potentially mitigating overcontrol bias inherent in previous studies using conventional survival models.

## Methods

### Study design

In this longitudinal study, we followed a cohort of beneficiaries from a large South African private medical insurance scheme. We analysed outpatient, laboratory, hospital and drug claims from the medical insurance scheme and linked these with beneficiary vital status information from the National Population Register.

### Study population

We included beneficiaries aged 18 years or older, who had health insurance coverage at any point, between 1 January 2011 and 15 March 2020, the beginning of the COVID-19 pandemic in South Africa. We excluded beneficiaries with unknown sex, or age, MACE on the first day of follow-up, and those whose vital status could not be ascertained because they did not have civil identification numbers and could not be linked to the National Population Register.

### Measures

We defined the study measures based on the International Classification of Diseases, 10th Revision (ICD-10) diagnoses from outpatient and hospital claims, hospital procedure codes and medication claims. Hospital procedures were coded according to the Current Procedural Terminology (CPT) and medication claims according to the Anatomical Therapeutic Chemical (ATC) classification system.

The primary outcome was a three-point MACE (Bosco *et al.*, 2021), which included ICD-10 diagnoses of either myocardial infarction (I21–I22), stroke (I60–I61, I63–I64 and H34.1) or hospitalization for unstable angina (I20) or hospital procedure codes for revascularization (CPT 33,503–33,572 and 92,920–92,984). The complete list of ICD-10 and CPT codes is shown in the appendix (Tables S1 and S2). In sensitivity analysis, we considered a two-point MACE (MACE2) consisting of myocardial infarction or stroke and a four-point MACE (MACE4) consisting of myocardial infarction, stroke, hospitalization for unstable angina or revascularization and heart failure (Table S1) as outcomes. We also considered individual MACE components such as stroke (ischaemic, bleeding or unspecified) and acute coronary syndrome (myocardial infarction or hospitalization due to unstable angina and revascularization procedures) in sensitivity analyses. A single claim code was considered evidence of the outcome.

The primary exposure was at least one ICD-10 diagnosis of PTSD (F43.1). We considered the following psychiatric comorbidities that can increase the risk of both PTSD (Brewin *et al.*, 2000; Ozer *et al.*, 2003; Sareen, 2014) and CVD (Akasaki and Ohishi, 2020; Lambert *et al.*, 2022; Nambiema *et al.*, 2023; Shao *et al.*, 2020): substance use disorders (F10–F16 and F18–F19), major depressive disorder (F32–F33), psychotic disorders (F20–F29) and sleep disorders (F51 and G47). Additionally, we considered antipsychotic medication as a risk factor for CVD (De Hert *et al.*, 2018). Antipsychotic medication was defined based on ATC codes for psychotic medication (N05A).

We considered the following CVD risk factors: hypertension, diabetes mellitus, dyslipidaemia and HIV. Hypertension was defined based on ICD-10 codes for hypertensive disease (I10-I13, I15, H35.0 and I67.4), evidence of use of medication used to treat hypertension (i.e. certain diuretics, beta-blockers or drug combinations) or at least two elevated systolic (≥140 mmHg) or diastolic (≥90 mmHg) blood pressure measurements (Appendix, Table S3). Diabetes mellitus was defined using the ICD-10 codes for diabetes (E10–E14, H28, H36, M14.2, M14.6, G59.0, G63.2 or G99.0), evidence of use of medications used for diabetes control (ATC codes A10) or at least two abnormal laboratory results of HbA1c ≥6.5% (≥48 mmol/L), fasting blood glucose ≥7 mmol/L or random blood glucose ≥11.1 mmol/L (American Diabetes, 2021) (Appendix, Table S4). Dyslipidaemia was identified with the ICD-10 codes E78.0–E78.5, evidence of the use of lipid-modifying medication (ATC codes C10) or at least two abnormal lipid measurements (High-Density Lipoprotein [HDL] cholesterol <1 mmol/L, Low-Density Lipoprotein [LDL] cholesterol >4.1 mmol/L or total cholesterol >6.2 mmol/L) (Arnett *et al.*, 2019) (Appendix, Table S5). We considered persons to be HIV positive if they had received an HIV viral load, CD4 cell count or a positive confirmatory HIV test, an ICD-10 diagnosis for HIV (B20–B24, Z21, R75 and O98.7) or antiretroviral medication for treating HIV, excluding medication commonly used in pre- or post-exposure prophylaxis (Appendix, Table S6). Self-identified ethnic population groups were defined as Black African, Indian/Asian, mixed ancestry, white or unknown. We included beneficiaries’ age as a continuous variable. Psychiatric and physical health conditions were modelled as time-varying covariates. Individuals were considered exposed from the date of the first diagnosis of the respective condition.

### Statistical analysis

In survival analyses, we followed beneficiaries from the start of insurance coverage, their 18th birthday or 1 January 2011 (whichever occurred last) until the event of interest (e.g., the MACE), or censoring events such as death or end of insurance coverage (whichever occurred first), for a maximum of 8 years.

We estimated the cumulative incidence of PTSD and 95% CIs by sex considering death as a competing event (Gooley *et al.*, 1999). Next, we calculated adjusted hazard ratios (aHRs) for hypothesized predictors of PTSD using a Cox proportional hazards model, stratified by population group and adjusted for age group, sex and HIV status.

To assess the association between PTSD and MACE and evaluate the influence of potential confounders and mediators, we fitted three adjusted Cox proportional hazards models: model 1 adjusted for HIV and sociodemographic characteristics (age, sex and population group); model 2 adjusted for HIV, sociodemographic characteristics and psychiatric comorbidity (substance use disorders, psychotic, major depressive disorder and sleep disorders); model 3 adjusted for HIV, sociodemographic characteristics, psychiatric comorbidity and CVD risk factors (diabetes mellitus, dyslipidaemia and hypertension). We fitted Cox models for both sexes, separate models for male and female beneficiaries and tested for interactions between sex and PTSD. In sensitivity analysis, we fitted model 2 with alternative MACE definitions (MACE2 and MACE4) and MACE components separately (stroke and acute coronary syndrome). We did not consider secondary MACE (repeated events). Beneficiaries exited the study at their first MACE. This analytic approach ensures that the PTSD diagnoses occurred before the MACE. We fitted Cox models to examine the association between PTSD and CVD risk factors. We checked the proportional hazard assumption using log–log plots and estimating Schoenfeld residuals after fitting the Cox models. We stratified Cox models by variables not satisfying the proportional hazards assumption.

We used modern doubly robust causal inference methods, specifically longitudinal targeted maximum likelihood estimation (Lendle *et al.*, 2017; Schomaker *et al.*, 2019), to estimate the marginal risks of MACE over 8 years of follow-up from the start of insurance coverage, beneficiaries’ 18th birthday, or 1 January 2011, whichever occurred last. We modelled two scenarios: continuous PTSD presence throughout follow-up for all beneficiaries and the counterfactual, no PTSD presence for any beneficiary. We divided beneficiaries’ follow-up into 16 six-monthly intervals and assessed confounders, mediators and the outcome at the end of each interval. We adjusted for measured time-varying confounding by HIV (Tang *et al.*, 2020), CVD risk factors, and psychiatric comorbidity and antipsychotic medication. Results are presented as absolute risks, risk differences and risk ratios with 95% CIs. To avoid model misspecification in longitudinal targeted maximum likelihood estimation, we fitted models for the conditional expectation of both MACE and PTSD in each 6-month follow-up period using super learning. This data-adaptive approach combines different modelling approaches to minimize the expected prediction error (estimated via cross-validation). Our learner sets included logistic (additive) regression models (with penalized splines and optional interactions), Bayesian logistic regression models and multivariate adaptive regression splines, after prior variable screening with Least Absolute Shrinkage and Selection Operator (LASSO) (Tibshirani, 1996) and Cramer’s V (Heumann and Shalabh, 2016). Due to computational constraints, we split the total sample randomly into two 50% subsets, executed longitudinal targeted maximum likelihood estimation on each and combined the results.

Statistical analyses were performed using Stata (Version 16. College Station, TX: StataCorp) and R 4.1.2 (R Foundation for Statistical Computing, Vienna, Austria). Causal inference analysis was performed on UBELIX (http://www.id.unibe.ch/hpc), the high-performance computing cluster at the University of Bern.

### Ethical considerations

The Ethics Committee of the University of Cape Town in South Africa and the Cantonal Ethics Committee in Bern, Switzerland, granted permission for the analysis of this database.

## Results

### Study population

The database included 1,537,056 beneficiaries who had insurance coverage at any point between 1 January 2011 and 15 March 2020. We excluded 527,943 (34.3%) beneficiaries, including 438,759 (28.5%) children and adolescents under the age of 18, 70,062 (4.6%) persons who could not be linked to the National Population Register, 9,928 (0.6%) persons with unknown sex, 9,108 (0.6%) persons with unknown age and 86 who had an MACE on the first day of follow-up (Figure S1).

### Characteristics of the study population

We followed 1,009,113 beneficiaries over a median duration of 3 years (IQR 1.1–6.0). During follow-up, 12,662 (1.3%) beneficiaries were diagnosed with PTSD and 39,255 (3.9%) had an MACE (Table 1). Half of the study population (51.8%) were female, and the mean age was 39.3 years (SD 15.2). Hypertension was the most frequent CVD risk factor (29.0%) followed by dyslipidaemia (15.7%) and diabetes (12.4%). Of the study population, 8.2% had a positive HIV status. Major depressive disorder (13.9%) was the most frequently diagnosed mental health condition (Table 1).

**Table 1.** Characteristics of beneficiaries with and without PTSD diagnosis at the end of follow-up

	PTSD	No PTSD	Total
	*N* = 12,662 (1.3)	*N* = 996,451 (98.7)	*N* = 1,009,113 (100.0)
**Characteristics at baseline, n (%)**			
Age, mean (SD)	37.9 (12.1)	39.3 (15.2)	39.3 (15.2)
Sex			
Male	4,842 (38.2)	481,569 (48.3)	486,411 (48.2)
Female	7,820 (61.8)	514,882 (51.7)	522,702 (51.8)
Population group			
Black African	8,382 (66.2)	515,225 (51.7)	523,607 (51.9)
Mixed Ancestry	769 (6.1)	64,978 (6.5)	65,747 (6.5)
White	1,744 (13.8)	182,939 (18.4)	184,683 (18.3)
Indian/Asian	366 (2.9)	45,650 (4.6)	46,016 (4.6)
Unknown	1,401 (11.1)	187,659 (18.8)	189,060 (18.7)
**Characteristics at the end of follow-up**			
CVD risk factors			
Diabetes mellitus	2,187 (17.3)	123,116 (12.4)	125,303 (12.4)
Hypertension	5,441 (43.0)	287,038 (28.8)	292,479 (29.0)
Dyslipidaemia	2,705 (21.4)	156,179 (15.7)	158,884 (15.7)
HIV	2,259 (17.8)	80,725 (8.1)	82,984 (8.2)
Psychiatric comorbidity			
Substance use disorders	449 (3.5)	8,977 (0.9)	9,426 (0.9)
Psychotic disorders	193 (1.5)	4,046 (0.4)	4,239 (0.4)
Major depressive disorder	6,921 (54.7)	133,609 (13.4)	140,530 (13.9)
Sleep disorders	2,440 (19.3)	66,122 (6.6)	68,562 (6.8)
Antipsychotic medication	3,136 (24.8)	92,557 (9.3)	95,693 (9.5)
Major adverse cardiovascular event (MACE)^a^	746 (5.9)	38,509 (3.9)	39,255 (3.9)
Myocardial infarction	117 (0.9)	7,233 (0.7)	7,350 (0.7)
Stroke	399 (3.2)	19,768 (2.0)	20,167 (2.0)
Hospitalization for unstable angina or revascularization	354 (2.8)	18,638 (1.9)	18,992 (1.9)
**Follow-up time, years**			
Median (IQR)	6.2 (3.2−8.0)	3.0 (1.1−6.0)	3.0 (1.1−6.0)

Abbreviations: PTSD, post-traumatic stress disorder; SD, standard deviation; CVD, cardiovascular disease; HIV, human immunodeficiency virus; MACE, major adverse cardiovascular event; IQR, interquartile range.

a MACE: myocardial infarction, stroke or hospitalization for unstable angina/revascularization.

### The cumulative incidence of PTSD and associated factors

**Figure 1.**
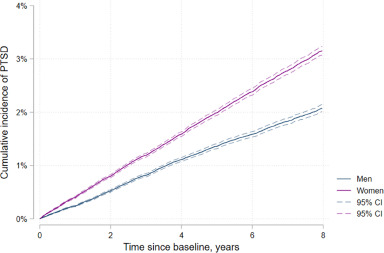
Cumulative incidence of PTSD by sex.

Abbreviations: PTSD, post-traumatic stress disorder; CI, confidence interval.

Figure 1 shows the cumulative incidence of PTSD by sex. After 8 years of follow-up, the cumulative incidence was 3.2% (95% CI 3.1–3.2) among females and 2.1% (95% CI 2.0–2.1) among males. Females (aHR 1.55, 95% CI 1.50–1.61), beneficiaries aged 35–44 years (aHR 1.13, 95% CI 1.07–1.18) and people living with HIV (aHR 1.34, 95% CI 1.27–1.42) had a higher risk of PTSD (Appendix, Table S7).

### Association between PTSD and MACE

**Figure 2.**
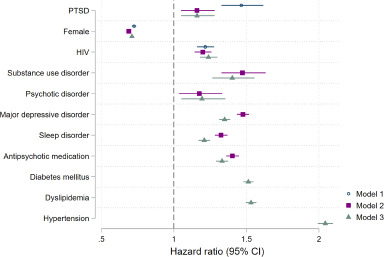
Factors associated with major adverse cardiovascular events^1^.

Abbreviations: PTSD, post-traumatic stress disorder; HIV, human immunodeficiency virus; CI, confidence interval. ^1^We considered the following major adverse cardiovascular events: myocardial infarction, stroke or hospitalization for unstable angina/revascularization. Model 1 was adjusted for PTSD, sex, HIV, age and population group. Model 2 was adjusted for PTSD, sex, HIV, age, population group, substance use disorder, psychotic disorders, major depressive disorder, use of antipsychotic medication and sleep disorder. Model 3 was adjusted for PTSD, sex HIV, age, population group, substance use disorder, psychotic disorder, major depressive disorder, use of antipsychotic medication, sleep disorder, diabetes, dyslipidaemia and hypertension.

After adjustment for sex, HIV status, age and population group (model 1), PTSD was associated with an increased risk of MACE (aHR 1.46, 95% CI 1.33–1.62). In a model adjusted for sex, HIV status, age, population group, psychiatric comorbidities and antipsychotic medication (model 2), the increase in risk was attenuated (aHR 1.16, 95% CI 1.05–1.28). Additional adjustment for CVD risk factors (model 3) did not affect the association between PTSD and MACE (aHR 1.16, 95% CI 1.05–1.28) (Fig. 2, Appendix, Table S8). Results were similar in sensitivity analyses of associations between PTSD and MACE2 (model 2: aHR 1.17, 95% CI 1.04–1.32) and MACE4 (model 2: aHR 1.14, 95% CI 1.05–1.24) (Appendix, Table S8). Associations between PTSD and MACE were similar among male and female beneficiaries (Appendix, Figure S2), and the test for an interaction between PTSD and sex was not significant in model 1 (*P* = 0.317) and model 2 (*P* = 0.244). PTSD was associated with an increased risk of incident stroke (aHR 1.18, 95% CI 1.03–1.35) and acute coronary syndrome (aHR 1.22, 95% CI 1.06–1.39) in models adjusted for sex, HIV status, age, population group, and psychiatric comorbidities and use of antipsychotic medication (model 2) (Figure S3).

### Association between PTSD and CVD risk factors

PTSD diagnosis was associated with an increased incidence of hypertension (aHR 1.23, 95% CI 1.17–1.29), diabetes mellitus (aHR 1.12, 95% 1.05–1.20) and dyslipidaemia (1.32, 95% 1.25–1.40) after adjusting for age, sex, population group and psychiatric comorbidities.

### Risk of MACE under PTSD and no PTSD

**Figure 3.**
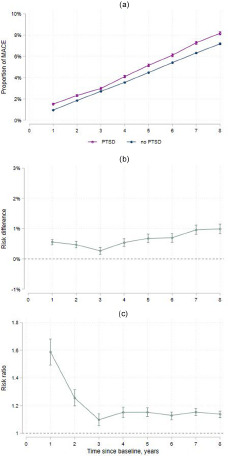
Major adverse cardiovascular events under PTSD and no PTSD.

Abbreviations: PTSD, post-traumatic stress disorder; MACEs, major adverse cardiovascular events.

Using longitudinal targeted maximum likelihood estimation, we calculated an absolute risk of MACE after 8 years of follow-up of 7.2% under no PTSD and 8.2% under PTSD (Fig. 3, panel A). After year 3, the risk difference between PTSD and no PTSD increased from 0.3% (95% CI 0.2–0.4) to 1.0% (95% CI 0.8–1.2) in year 8 (Fig. 3, panel B). Risk ratios decreased from 1.59 (95% CI 1.49–1.68) in year 1 to 1.14 (95% CI 1.11–1.16) in year 8 (Fig. 3, panel C).

## Discussion

In this cohort study of over one million South African medical insurance beneficiaries, we demonstrated an increased risk of MACE among individuals diagnosed with PTSD compared to those without it, using both traditional survival analysis and modern causal inference methods. In survival analysis, adjusted for sociodemographic factors, HIV and psychiatric comorbidities, beneficiaries with a PTSD diagnosis experienced a 16% increase in MACE risk compared to beneficiaries without PTSD. The causal inference analysis showed a 14% increase in MACE risk for the PTSD scenario compared to the non-PTSD scenario after the eighth year of follow-up.

There are two potential causal mechanisms that might explain the increased risk of CVD in individuals with PTSD. The first mechanism is psychological and behavioural, with PTSD increasing the risk of depression, substance misuse and sleep disorders and these factors leading to unhealthy lifestyle choices such as reduced physical activity and poor dietary habits. These behaviours can subsequently contribute to the development of CVD risk factors including hypertension, diabetes and high cholesterol, which may ultimately result in CVD (Edmondson and von Kanel, 2017; Scherrer *et al.*, 2019). The second mechanism is pathophysiological, as PTSD can impact various physiological processes that may directly cause cardiovascular events, such as myocardial infarction, unstable angina or stroke. These processes include systemic inflammation, dysregulation of the hypothalamic–pituitary–adrenal axis and imbalances in the autonomic nervous system (Edmondson and von Kanel, 2017). In our study, we evaluated the potential contribution of CVD risk factors, specifically hypertension, diabetes and dyslipidaemia. Using Cox models adjusted for sex, HIV status, age, population group and psychiatric comorbidities, we found a 16% increased risk of MACE in individuals diagnosed with PTSD. After further adjustments for CVD risk factors, this association remained unchanged. This finding suggests that in our study population the elevated MACE risk in individuals with PTSD occurs independent of traditional CVD risk factors. Physiological changes induced by PTSD might play a significant role in driving the increased risk of MACE observed in our study.

With the exception of few studies from Sweden (Song *et al.*, 2019), Denmark (Gradus *et al.*, 2015) and Taiwan (Chen *et al.*, 2015), most previous research in this field was conducted has been among males from the US and has primarily focused on war- and terror-related trauma among veterans and World Trade Centre survivors. In contrast, our study extends this knowledge by using a general population sample likely exposed to a variety of traumatic experiences, which are prevalent in the context of South Africa. Common traumas in South Africa are physical or sexual violence, the death of a loved one, witnessing traumatic incidents and accidents (Atwoli *et al.*, 2013). Our findings not only reinforce and complement the prevailing evidence but also extend our understanding the implications of PTSD in diverse, trauma-exposed, and middle-income settings. The consistency of our findings with prior research strengthens the credibility of the elevated CVD risk in individuals with PTSD and underscores their wide applicability across diverse countries, populations and trauma exposures.

The relationship between PTSD and psychiatric comorbidities is complex and bi-directional. Psychiatric comorbidities like depression, substance use disorders, sleep disturbance and generalized anxiety disorder can act as important confounders in the relationship between PTSD and CVD, making adjustments for their effects crucial. On the other hand, psychiatric comorbidities can also mediate the relationship between PTSD and CVD (Scherrer *et al.*, 2019). In this situation, using traditional survival models to adjust for psychiatric comorbidities can lead to overadjustment bias (Mansournia *et al.*, 2017; Schisterman *et al.*, 2009). Despite this potential bias, much of the previous research has adjusted for psychiatric comorbidity using traditional survival models. For instance, a recent meta-analysis of 14 studies investigating the relationship between PTSD and myocardial infarction included nine studies that adjusted for depression (Jacquet-Smailovic *et al.*, 2022). To overcome this issue, we implemented modern causal inference methods, which can adjust for time-varying confounding affected by prior exposure without blocking the causal pathway from exposure to outcome (Petersen *et al.*, 2014). With these methods, we could appropriately adjust for important potential confounders like depression, substance use disorders, psychotic disorders and sleep disturbance. We could, however, not adjust for other anxiety disorders. The common symptoms between PTSD and other anxiety disorders complicate differential diagnosis. In our data, many patients initially diagnosed with an unspecific or another anxiety disorder were later accurately diagnosed with PTSD. Consequently, adjusting for other anxiety disorders would lead to overadjustment. From a clinical standpoint, it may also be questionable whether adjusting for psychiatric comorbidities, like depression and other anxiety disorders, to estimate an independent effect of PTSD on cardiovascular outcomes is justified. Psychiatric comorbidity is the rule, rather than the exception in PTSD, with epidemiologic studies demonstrating comorbidity in over 75% of patients with PTSD, with depression the most frequent comorbid disorder (Brady *et al.*, 2000; Kessler, 2017). Psychiatric comorbidity is also associated with chronicity, greater functional impairment and worse outcomes, including poorer response to treatment (Brady *et al.*, 2000, Flory and Yehuda, 2015). There is also evidence to suggest that individuals with comorbid depression may represent a subtype of PTSD, with shared negative affectivity (Contractor *et al.*, 2020; Flory and Yehuda, 2015). Since PTSD, depression and other anxiety disorders share common symptoms (Angelakis and Nixon, 2015; Flory and Yehuda, 2015) and comorbidity may reflect severity of PTSD, such adjustment could potentially be seen as overadjusting for PTSD severity. Given that we used ICD10 diagnoses from administrative data, this issue might be further complicated by potential misdiagnoses of PTSD as depression due to symptom overlap.

This study has important implications for clinical practice. Despite the growing evidence of the impact of mental disorders on physical comorbidity and premature mortality (Ruffieux *et al.*, 2023), mental healthcare in many settings remains underfunded and segmented into silos of mental and physical healthcare (O’Connor *et al.*, 2023). To address the physical healthcare needs of individuals with mental illnesses, the World Health Organization, along with international experts, advocates for integrated care models that combine mental health services with physical healthcare (O’Connor *et al.*, 2023). It is crucial for health professionals to be informed about the potential cardiovascular implications of PTSD to ensure comprehensive care including management of CVD risk factors.

Key strengths of our study include our distinctive study population and our analytic approach. Our sample is drawn from a population exposed to a wide spectrum of traumatic experiences and females. Our second major strength is the use of modern causal inference methods, which enabled us to overcome methodological limitations of previous research. The use of modern causal inference methods has several benefits over traditional survival analysis methods. First, the causal inference methods allow to adjust for time-dependent confounding by psychiatric comorbidity and CVD risk factors without blocking the causal path from PTSD to MACE (Hernán and Robins, 2020). Second, our chosen estimands (i.e. causal risks) prevent potential survivor bias inherent in traditional survival analysis due to conditioning on being event-free under PTSD/no PTSD at each time point in the definition of the hazard (Young *et al.*, 2020). Third, longitudinal targeted maximum likelihood estimation is a doubly robust method that incorporates machine learning procedures to reduce the risk of bias due to model misspecification (Van der Laan and Rose, 2011).

Our results should be interpreted considering the following limitations. First, our study’s results, focused on South African private medical insurance beneficiaries, are not representative of the general population of South Africa since only 15% of the populace has such coverage (Day and Gray, 2013). This insured group generally has a higher socioeconomic status (Gordon *et al.*, 2020) and likely lower exposure to trauma and poor health outcomes than those using the public health system. Second, we used medical insurance scheme data to determine the presence of MACE, PTSD and physical and psychiatric comorbidities. Administrative data limitations may include inaccuracy and misclassification. We based PTSD and psychiatric comorbidity presence on ICD-10 diagnoses from outpatient and hospital claims, possibly overlooking undiagnosed cases or those not seeking care. Our MACE definition depended on ICD-10 diagnoses and procedure codes, potentially missing cases who died before medical attention. Third, our dataset lacked information on socioeconomic factors, other CVD risk factors or behaviours, or pre-insurance medical history, which could confound results. Moreover, the lack of pre-insurance medical history could be an explanation for the relatively high risk ratio of the absolute risks in year 1. Factors like employment, education and neighbourhood characteristics impact CVD (Schultz *et al.*, 2018), PTSD vulnerability (DiGangi *et al.*, 2013) and control of CVD risk factors (Rosengren *et al.*, 2019). Our study group, being private insurance members, was relatively homogeneous with regard to socioeconomic status. Still, residual confounding from socioeconomic differences among these members may still impact our estimates. Fourth, a notable limitation of our study is the exclusion of cardiovascular death in our definition of MACE. This exclusion stems from our lack of access to detailed data regarding the exact causes of death among the beneficiaries. Consequently, we may have overlooked MACE events in individuals who succumbed to an MACE-related cause but died before they could be diagnosed with an MACE or underwent a revascularization procedure. This limitation leads to an underestimation of the MACE prevalence in our study. Fifth, our dataset did not contain information on traumatic events, and as such, we could not compare the MACE risk between individuals exposed to traumatic events with and without subsequent PTSD. Consequently, we were unable to determine whether the increased MACE risk is a consequence of the traumatic event itself or the psychological sequelae that characterize PTSD.

## Conclusion

Our study provides empirical support for an increased risk of MACE among males and female beneficiaries with PTSD in South Africa, a population exposed to a range of traumas that extend beyond war-related experiences. These findings highlight the importance of monitoring cardiovascular risk among individuals diagnosed with PTSD and support integrated care models that combine mental health services with physical healthcare.

## Supporting information

Mesa-Vieira et al. supplementary material Mesa-Vieira et al. supplementary material

## Data Availability

Data were obtained from the International Epidemiology Databases to Evaluate AIDS-Southern Africa (IeDEA-SA). Due to legal and ethical restrictions, the data cannot be made publicly available. For inquiries about the data, readers can contact IeDEA-SA through the online form available at https://www.iedea-sa.org/contact-us/.

## Supplementary material

The supplementary material for this article can be found at https://doi.org/10.1017/S2045796024000052.
