# Supplementary material for: Post-traumatic stress disorder as a risk factor for major adverse cardiovascular events: a cohort study of a South African medical insurance scheme
Source: Epidemiol Psychiatr Sci. 2024 Feb 5;33:e5. doi: 10.1017/S2045796024000052 (PMC10894700; doi:10.1017/S2045796024000052)
Supplement: Mesa-Vieira et al. supplementary material [file S2045796024000052sup001.docx]

**Supplementary material**

Figure S1. Flow chart for the selection of study participants

**
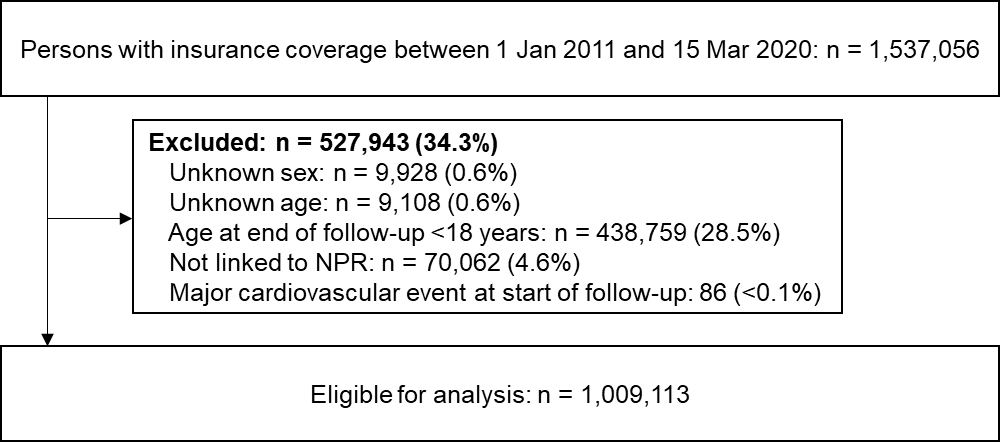
**

NPR=National Population Register

Figure S2. Adjusted hazard ratios for major adverse cardiovascular events^1^ by sex

Abbreviations: PTSD, post-traumatic stress disorder; HIV, human immunodeficiency virus.

^1^ We considered the following major adverse cardiovascular events: myocardial infarction, stroke, or hospitalization for unstable angina/revascularization

Model 1 was adjusted for PTSD, sex, HIV, age, and population group. Model 2 was adjusted for PTSD, sex, HIV, age, population group, substance use disorder, psychotic disorder, major depressive disorder, sleep disorders, and use of antipsychotic medication

Figure S3. Adjusted hazard ratios for stroke^1^ and acute coronary syndromes^2^

Abbreviations: PTSD, post-traumatic stress disorder; HIV, human immunodeficiency virus.

^1^ We considered the following stroke: bleeding stroke, ischaemic stroke and unspecified stroke

^2^ We considered the following acute coronary syndromes: ST-elevation myocardial infarction, non-ST-elevation myocardial infarction, unspecified myocardial infarction and hospitalization for unstable angina or revascularization

Model 2 was adjusted for PTSD, sex, HIV, age, population group, substance use disorder, psychotic disorder, major depressive disorder, sleep disorder, and the use of antipsychotic medication.

Table S1: List of International Classification of Diseases, 10^th^ Revision (ICD-10) codes of acute coronary syndromes, stroke, and heart failure

| **Condition** | **ICD-10**  **Code** |
| --- | --- |
| **Acute coronary syndromes** |  |
| Unstable angina | I20.0 |
| Acute ST-elevation myocardial infarction |  |
| Transmural infarct of anterior wall | I21.0 |
| Transmural infarct of inferior wall | I21.1 |
| Transmural infarct of other sites | I21.2 |
| Transmural infarct of unspecified sites | I21.3 |
| Subsequent (<4w) infarction of anterior wall | I22.0 |
| Subsequent (<4w) infarction of inferior wall | I22.1 |
| Subsequent (<4w) infarction of other sites | I22.8 |
| Subsequent (<4w) infarction of unspecified sites | I22.9 |
| Acute non-ST-elevation myocardial infarction |  |
| Acute subendocardial myocardial infarction | I21.4 |
| Unspecified myocardial infarction |  |
| Acute myocardial infarction, unspecified | I21.9 |
| **Stroke** |  |
| Haemorrhagic stroke |  |
| Subarachnoid haemorrhage from carotid siphon and bifurcation | I60.0 |
| Subarachnoid haemorrhage from middle cerebral artery | I60.1 |
| Subarachnoid haemorrhage from anterior communicating artery | I60.2 |
| Subarachnoid haemorrhage from posterior communicating artery | I60.3 |
| Subarachnoid haemorrhage from basilar artery | I60.4 |
| Subarachnoid haemorrhage from vertebral artery | I60.5 |
| Subarachnoid haemorrhage from other intracranial arteries | I60.6 |
| Subarachnoid haemorrhage from intracranial artery, unspecified | I60.7 |
| Other subarachnoid haemorrhage | I60.8 |
| Subarachnoid haemorrhage, unspecified | I60.9 |
| Intracerebral haemorrhage in hemisphere, subcortical | I61.0 |
| Intracerebral haemorrhage in hemisphere, cortical | I61.1 |
| Intracerebral haemorrhage in hemisphere, unspecified | I61.2 |
| Intracerebral haemorrhage in brain stem | I61.3 |
| Intracerebral haemorrhage in cerebellum | I61.4 |
| Intracerebral haemorrhage, intraventricular | I61.5 |
| Intracerebral haemorrhage, multiple localized | I61.6 |
| Other intracerebral haemorrhage | I61.8 |
| Intracerebral haemorrhage, unspecified | I61.9 |
| Ischaemic stroke |  |
| Cerebral infarction due to thrombosis of precerebral arteries | I63.0 |
| Cerebral infarction due to embolism of precerebral arteries | I63.1 |
| Cerebral infarction due to unspecified occlusion or stenosis of precerebral arteries | I63.2 |
| Cerebral infarction due to thrombosis of cerebral arteries | I63.3 |
| Cerebral infarction due to embolism of cerebral arteries | I63.4 |
| Cerebral infarction due to unspecified occlusion or stenosis of cerebral arteries | I63.5 |
| Other cerebral infarction | I63.8 |
| Cerebral infarction, unspecified | I63.9 |
| Central retinal artery occlusion | H34.1 |
| Unspecified stroke |  |
| Stroke, not specified as haemorrhage or infarction | I64 |
| **Heart failure** |  |
| Hypertensive heart disease with (congestive) heart failure | I11.0 |
| Heart failure | I50 |
| Cardiac insufficiency or heart failure following cardiac surgery | I97.1 |

Table S2: List of Current Procedural Terminology (CPT) codes for coronary revascularisation procedures

| **Procedure** | **CPT code** |
| --- | --- |
| Coronary revascularisation procedures |  |
| Coronary artery graft | 33503 |
| Coronary artery graft | 33504 |
| Coronary artery bypass graft, vein, single | 33510 |
| Coronary artery bypass graft, vein, two | 33511 |
| Coronary artery bypass graft, vein, three | 33512 |
| Coronary artery bypass graft, vein, four | 33513 |
| Coronary artery bypass graft, vein, five | 33514 |
| Coronary artery bypass graft, vein, six or more | 33516 |
| Coronary artery bypass graft, artery-vein, single | 33517 |
| Coronary artery bypass graft, artery-vein, two | 33518 |
| Coronary artery bypass graft, artery-vein, three | 33519 |
| Coronary artery bypass graft, artery-vein, four | 33521 |
| Coronary artery bypass graft, artery-vein, five | 33522 |
| Coronary artery bypass graft, artery-vein, six or more | 33523 |
| Coronary artery, bypass/reoperation | 33530 |
| Coronary artery bypass graft, arterial, single | 33533 |
| Coronary artery bypass graft, arterial, two | 33534 |
| Coronary artery bypass graft, arterial, three | 33535 |
| Coronary artery bypass graft, arterial, four or more | 33536 |
| Open coronary endarterectomy | 33572 |
| Percutaneous transluminal coronary angioplasty, single major coronary artery or branch | 92920 |
| Percutaneous transluminal coronary angioplasty, each add. branch of maj. coronary artery | 92921 |
| Percutaneous transluminal coronary atherectomy, with coronary angioplasty when performed; single major coronary artery or branch | 92924 |
| Percutaneous transcatheter placement of intracoronary stent(s), with coronary angioplasty when performed; single major coronary artery or branch | 92928 |
| Percutaneous transcatheter placement of intracoronary stent(s), with coronary angioplasty when performed; each additional branch of a major coronary artery | 92929 |
| Percutaneous transluminal coronary atherectomy, with intracoronary stent, with coronary angioplasty when performed; single major coronary artery or branch | 92933 |
| Percutaneous transluminal coronary atherectomy, with intracoronary stent, with coronary angioplasty when performed; each additional branch of a major coronary artery | 92934 |
| Percutaneous transluminal revascularisation of or through coronary artery bypass graft (internal mammary, free arterial, venous), any combination of intracoronary stent, atherectomy and angioplasty, including distal protection when performed; single vessel | 92937 |
| Percutaneous transluminal revascularisation of or through coronary artery bypass graft (internal mammary, free arterial, venous), any combination of intracoronary stent, atherectomy and angioplasty, including distal protection when performed; each additional branch subtended by the bypass graft | 92938 |
| Percutaneous transluminal revascularisation of acute total/subtotal occlusion during acute myocardial infarction, coronary artery or coronary artery bypass graft, any combination of intracoronary stent, atherectomy and angioplasty, including aspiration thrombectomy when performed, single vessel | 92941 |
| Percutaneous transluminal revascularisation of chronic total occlusion, coronary artery, coronary artery branch, or coronary artery bypass graft, any combination of intracoronary stent, atherectomy and angioplasty; single vessel | 92943 |
| Percutaneous transluminal revascularisation of chronic total occlusion, coronary artery, coronary artery branch, or coronary artery bypass graft, any combination of intracoronary stent, atherectomy and angioplasty; each additional coronary artery, coronary artery branch, or bypass graft | 92944 |
| Percutaneous transluminal coronary thrombectomy | 92973 |
| Insert intracoronary stent | 92980 |
| Insert intracoronary stent | 92981 |
| Coronary artery dilation | 92982 |
| Coronary artery dilation | 92984 |

Table S3: List of diagnoses, medications, and test results indicative of hypertension

| **Diagnoses** | **ICD10 code** |
| --- | --- |
| Essential hypertension | I10 |
| Hypertensive heart disease | I11 |
| Hypertensive renal disease | I12 |
| Hypertensive heart and renal disease | I13 |
| Secondary hypertension | I15 |
| Hypertensive retinopathy | H35.0 |
| Hypertensive encephalopathy | I67.4 |
| **Drug** | **ATC code** |
| Low-ceiling diuretics (thiazides) | C03A |
| Low-ceiling diuretics (non-thiazides) | C03B |
| Low-ceiling diuretics in combination with potassium-sparing agents | C03EA |
| Beta-blockers combined with thiazides | C07B |
| Beta-blockers with “other” diuretics | C07C |
| Beta-blockers with thiazide diuretic with "other" diuretics | C07D |
| Calcium channel blockers in combination with diuretics | C08G |
| ACE-inhibitors with diuretics | C09BA |
| Angiotensin II receptor-blockers with diuretics | C09DA |
| Valsartan + amlodipine + hydrochlorothiazide | C09DX01 |
| Olmesartan + amlodipine + hydrochlorothiazide | C09DX03 |
| Candesartan + amlodipine + hydrochlorothiazide | C09DX06 |
| Aliskiren + hydrochlorothiazide | C09XA52 |
| Aliskiren + amlodipine + hydrochlorothiazide | C09XA54 |
| Rosuvastatin + perindopril + indapamide | C10BX13 |
| **Clinical test** | **Value** |
| Systolic blood pressure | ≥140mmHg |
| Diastolic blood pressure | ≥90mmHg |

Table S4: List of diagnoses, medications, and laboratory test results indicative of diabetes mellitus

| **Diagnoses** | **ICD10 code** |
| --- | --- |
| Type 1 diabetes mellitus | E10 |
| Type 2 diabetes mellitus | E11 |
| Malnutrition-related diabetes mellitus | E12 |
| Other specified diabetes mellitus | E13 |
| Unspecified diabetes mellitus | E14 |
| Diabetic cataract | H28.0 |
| Diabetic retinopathy | H36.0 |
| Diabetic arthropathy | M14.2 |
| Diabetic neuropathic arthropathy | M14.6 |
| Diabetic mononeuropathy | G59.0 |
| Diabetic polyneuropathy | G63.2 |
| Diabetic autonomic neuropathy | G99.0 |
| **Drug** | **ATC code** |
| Drugs used in diabetes | A10 |
| **Laboratory test** | **Value** |
| HbA1c | ≥6.5 % (≥48mmol/L) |
| Fasting blood glucose | ≥7.0 mmol/L (~126 mg/dL) |
| Random blood glucose | ≥11.1 mmol/L (~200 mg/dL) |

Table S5: List of diagnoses, medications, and test results indicative of dyslipidaemia

| **Diagnoses** | **ICD10 code** |
| --- | --- |
| Pure hypercholesterolaemia | E78.0 |
| Pure hyperglyceridaemia | E78.1 |
| Mixed hyperlipidaemia | E78.2 |
| Hyperchylomicronaemia | E78.3 |
| Other hyperlipidaemia | E78.4 |
| Hyperlipidaemia, unspecified | E78.5 |
| **Drug** | **ATC code** |
| Lipid-modifying agents | C10 |
| **Laboratory test** | **Value** |
| HDL-cholesterol | <1 mmol/L (~40 mg/dL) |
| LDL-cholesterol | >4.1 mmol/L (~160 mg/dL) |
| Total cholesterol | >6.2 mmol/L (~240 mg/dL) |

Table S6: List of diagnoses, medications, and test results indicative of HIV

| **Diagnoses** | **ICD10 code** |
| --- | --- |
| Human immunodeficiency virus (HIV) disease | B20-B24 |
| Asymptomatic HIV infection status | Z21 |
| Laboratory evidence of HIV | R75 |
| HIV disease complicating pregnancy, childbirth and the puerperium | O98.7 |
| **Antiretroviral medication for treating HIV** | **ATC code** |
| Protease inhibitors | J05AE |
| Nucleoside and nucleotide reverse transcriptase inhibitors | J05AF |
| Non-nucleoside reverse transcriptase inhibitors | J05AG |
| Integrase inhibitors | J05AJ |
| Antivirals for treatment of HIV infections, combinations | J05AR |
| **Antiretroviral medication used in pre- or post-exposure prophylaxis** | **ATC code** |
| Tenofovir disoproxil and emtricitabine (TDF/FTC) | J05AR03 |
| Tenofovir alafenamide (TAF) | J05AF13 |
| Emtricitabine (FTC) | J05AF09 |
| Lamivudine (3TC) | J05AF05 |
| **Laboratory test** | **Value** |
| Confirmatory HIV test | Positive |
| HIV viral load | Any |
| CD4 cell count | Any |

Table S7: List of antipsychotic medications

| **Antipsychotic medication** | **ATC code** |
| --- | --- |
| [Phenothiazines](https://en.wikipedia.org/wiki/Phenothiazine) with aliphatic side-chain | N05AA |
| Phenothiazines with [piperazine](https://en.wikipedia.org/wiki/Piperazine) structure | N05AB |
| Phenothiazines with [piperidine](https://en.wikipedia.org/wiki/Piperidine) structure | N05AC |
| [Butyrophenone](https://en.wikipedia.org/wiki/Butyrophenone) derivatives | N05AD |
| [Indole](https://en.wikipedia.org/wiki/Indole) derivatives | N05AE |
| [Thioxanthene](https://en.wikipedia.org/wiki/Thioxanthene) derivatives | N05AF |
| [Diphenylbutylpiperidine](https://en.wikipedia.org/wiki/Diphenylbutylpiperidine) derivatives | N05AG |
| [Diazepines](https://en.wikipedia.org/wiki/Diazepine), [oxazepines](https://en.wikipedia.org/wiki/Oxazepine), [thiazepines](https://en.wikipedia.org/wiki/Thiazepine) and [oxepines](https://en.wikipedia.org/wiki/Oxepine) | N05AH |
| [Benzamides](https://en.wikipedia.org/wiki/Benzamide) | N05AL |
| [Lithium](https://en.wikipedia.org/wiki/Lithium) | N05AN |
| Other antipsychotics | N05AX |

Table S8: Factors associated with post-traumatic stress disorder (PTSD)

|  | **HR (95% CI)** | **aHR**^1^ **(95% CI)** |
| --- | --- | --- |
| Age, years |  |  |
| 18-24 | 0.57 (0.53-0.62) | 0.59 (0.54-0.64) |
| 25-34 | 1.06 (1.01-1.12) | 0.99 (0.94-1.05) |
| 35-44 | 1.19 (1.13-1.25) | 1.13 (1.07-1.18) |
| 45-54 | 1.00 | 1.00 |
| 55-64 | 0.64 (0.60-0.68) | 0.68 (0.64-0.73) |
| 65-74 | 0.32 (0.28-0.36) | 0.39 (0.35-0.44) |
| 75+ | 0.26 (0.21-0.30) | 0.32 (0.27-0.38) |
| Sex |  |  |
| Male | 1.00 | 1.00 |
| Female | 1.50 (1.45-1.56) | 1.55 (1.50-1.61) |
| HIV status |  |  |
| Negative or unknown | 1.00 | 1.00 |
| Positive | 1.90 (1.80-2.00) | 1.34 (1.27-1.42) |

HIV, human immunodeficiency virus; HR, hazard ratio, aHR, adjusted hazard ratio, CI, confidence interval

^1^ Adjusted for age, sex, HIV status, and population group.

Table S9. Adjusted hazard ratios for factors associated with various definitions for major adverse cardiovascular events

|  | **MACE**^1^ | | | | **MACE2**^2^ | **MACE4**^3^ |
| --- | --- | --- | --- | --- | --- | --- |
|  | Unadjusted | Model 1^4^ | Model 2^4^ | Model 3^4^ | Model 2^4^ | Model 2^4^ |
|  | HR (95% CI) | aHR (95% CI) | aHR (95% CI) | aHR (95% CI) | aHR (95% CI) | aHR (95% CI) |
| Mental disorders |  |  |  |  |  |  |
| PTSD | 0.99 (0.89-1.09) | 1.46 (1.33-1.62) | 1.16 (1.05-1.28) | 1.16 (1.05-1.28) | 1.17 (1.04-1.32) | 1.14 (1.05-1.24) |
| Substance use disorders | 1.48 (1.33-1.63) |  | 1.47 (1.33-1.63) | 1.40 (1.26-1.56) | 1.54 (1.36-1.74) | 1.46 (1.33-1.60) |
| Psychotic disorders | 1.97 (1.74-2.23) |  | 1.17 (1.03-1.33) | 1.19 (1.05-1.35) | 1.33 (1.16-1.53) | 1.07 (0.96-1.20) |
| Major depressive disorder | 1.69 (1.65-1.74) |  | 1.48 (1.43-1.52) | 1.35 (1.31-1.39) | 1.51 (1.46-1.56) | 1.41 (1.38-1.44) |
| Sleep disorders | 2.14 (2.07-2.21) |  | 1.32 (1.28-1.37) | 1.21 (1.17-1.25) | 1.30 (1.25-1.35) | 1.32 (1.28-1.35) |
| Antipsychotic medication | 1.76 (1.71-1.81) |  | 1.40 (1.36-1.45) | 1.33 (1.29-1.37) | 1.53 (1.48-1.59) | 1.35 (1.31-1.38) |
| Sex |  |  |  |  |  |  |
| Male | 1.00 | 1.00 | 1.00 | 1.00 | 1.00 | 1.00 |
| Female | 0.81 (0.80-0.83) | 0.73 (0.71-0.74) | 0.69 (0.68-0.70) | 0.71 (0.70-0.73) | 0.74 (0.72-0.75) | 0.81 (0.80-0.83) |
| Cardiovascular risk factors |  |  |  |  |  |  |
| Diabetes | 3.79 (3.71-3.88) |  |  | 1.51 (1.48-1.55) |  |  |
| Dyslipidemia | 5.09 (4.99-5.20) |  |  | 1.53 (1.50-1.57) |  |  |
| Hypertension | 5.71 (5.58-5.83) |  |  | 2.04 (1.99-2.10) |  |  |
| HIV | 0.60 (0.57-0.63) | 1.22 (1.16-1.28) | 1.20 (1.14-1.26) | 1.24 (1.18-1.30) | 1.36 (1.29-1.44) | 1.24 (1.19-1.28) |

MACE, major adverse cardiovascular event

PTSD, post-traumatic stress disorder

HIV, human immunodeficiency virus

^1^ MACE: myocardial infarction, stroke, or hospitalization for unstable angina/revascularization

^2^ MACE2: myocardial infarction, or stroke

^3^ MACE4: myocardial infarction, stroke, hospitalization for unstable angina/revascularization, or heart failure

^4^ Adjusted for all variables shown in the model, population group and age
